# Nystagmus and Vertigo During Aural Toilet Using Microsuction

**DOI:** 10.3390/audiolres15020033

**Published:** 2025-03-19

**Authors:** Chang-Hee Kim, Minho Jang, Taehee Kim, JiAh Kim, ChanEui Hong, Dong-Han Lee, Jung Eun Shin

**Affiliations:** Department of Otorhinolaryngology-Head and Neck Surgery, Konkuk University Medical Center, Konkuk University School of Medicine, Research Institute of Medical Science, Institute of Biomedical Science & Technology, Seoul 05030, Republic of Korea; 20200123@kuh.ac.kr (M.J.); 20200135@kuh.ac.kr (T.K.); 20210150@kuh.ac.kr (J.K.); 20210171@kuh.ac.kr (C.H.); 20200189@kuh.ac.kr (D.-H.L.); 20050055@kuh.ac.kr (J.E.S.)

**Keywords:** vertigo, nystagmus, microsuction, aural toilet

## Abstract

**Background/Objectives**: Aural toilet using microsuction is a common procedure in ENT clinics, and vertigo is a frequent complaint during this procedure. This study aimed to investigate the characteristics and incidence of microsuction-induced nystagmus and vertigo based on the appearance of the tympanic membrane (TM). **Methods**: In 85 patients with various TM appearances, microsuction-induced vertigo and nystagmus were assessed. **Results**: Microsuction elicited nystagmus in 95% (81 of 85) of patients and vertigo in 36% (31 of 85). The nystagmus direction was towards the ipsilateral ear in a bowing position and towards the contralateral ear in a leaning position. The proportion of patients who complained of rotatory vertigo was significantly higher in those with TM perforation, open cavity mastoidectomy, and adhesive otitis media (74%, 26 of 35) compared to those without TM perforation (10%, 5 of 50) (*p* < 0.001, X^2^ test). **Conclusions**: Aural toilet using microsuction commonly induces vertigo due to convection in the lateral semicircular canal endolymph caused by the cooling effect. While microsuction-induced nystagmus was observed in most patients, the incidence of vertigo varied depending on the TM condition. Clinicians should closely monitor patients for vertigo during the procedure, and methods to prevent microsuction-induced vertigo should be explored.

## 1. Introduction

Aural toilet using microsuction is a commonly performed procedure in ENT outpatient clinics [1]. With the aid of a microscope or otoendoscope, aural microsuction effectively and safely removes purulent discharge, foreign bodies, or ear wax [2]. In particular, the impaction of ear wax is encountered very often in ENT clinics, which can cause otalgia and/or conductive hearing loss. Aural microsuction is a good method of clearing the ear canal by removing the ear wax using a suction device guided by microscope visualization. It was reported that microsuction is the most commonly used method of ear wax removal at ENT clinics [3]. Microsuction is also advantageous for external and middle ear infections, as clearing discharge and debris enhances the absorption and distribution of topical medications. Despite its advantages, patients may experience discomfort, such as pain or tension, and various forms of dizziness, including rotatory vertigo or presyncope. Previous studies reported that vertigo is a frequent complaint during aural microsuction [3,4,5,6,7,8,9,10,11]. In some patients, microsuction-induced vertigo is so severe that the procedure cannot continue. Cooling of the middle ear cavity during microsuction has been suggested as the underlying cause of vertigo [4,7,9]. Vertigo is a sensation of spinning or dizziness, often caused by issues in the inner ear, vestibular nerve, or brain. It creates a false perception of movement, making a person feel as if they or their surroundings are rotating or tilting, even when they are stationary [12,13,14,15,16,17,18,19]. Nystagmus, which refers to a condition characterized by involuntary, repetitive eye movements that can be slow or fast and occur in various directions, is often accompanied by vertigo [20,21,22,23].

While microsuction-induced vertigo has been reported since the early 1900s and is frequently encountered in clinics, there have been no reports on the characteristics of nystagmus observed during these occurrences. This study investigates the characteristics of microsuction-induced nystagmus and the incidence of microsuction-induced vertigo or nystagmus based on the appearance of the tympanic membrane (TM).

## 2. Materials and Methods

Patients who underwent aural toilet using microsuction at our clinic between March 2022 and August 2024 were included in this study. Otoendoscopic examination was used to determine the need for microsuction, and nystagmus was observed using video Frenzel glasses. The patients required microsuction for various reasons, such as wet debris, purulent discharge, ear wax, or foreign body removal. A total of 85 patients (39 men and 46 women; mean age 58 ± 17 years) were enrolled. The patients were asked if they experienced rotatory vertigo during and after microsuction. Since the feeling of dizziness is a subjective symptom that can include various sensations such as lightheadedness, a sense of disorientation, imbalance or unsteadiness, and an illusory sensation of motion of either the self or the surroundings [24], patients were carefully asked to distinguish whether their dizziness was vertigo or another form of nonspecific dizziness. Those with congenital nystagmus, taking medication affecting nystagmus or dizziness, or have a history of vestibulopathy were excluded. Neurological examinations revealed no deficits, and spontaneous nystagmus before microsuction was another exclusion criterion.

**Figure 1:**
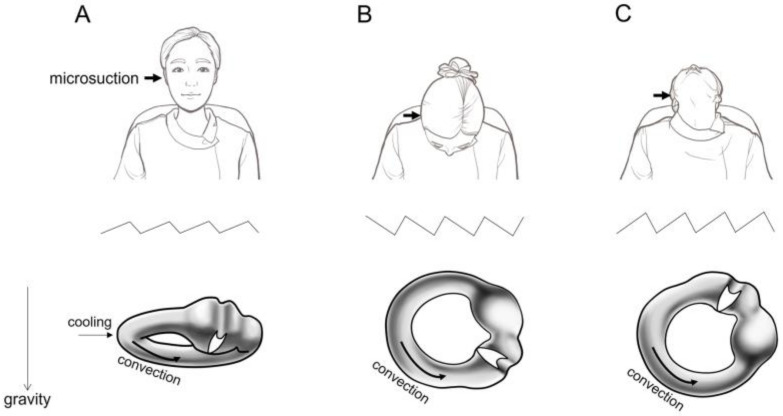
Representative picture of patient position and microsuction-induced nystagmus. (**A**) In an upright sitting position, microsuction for aural toilet was applied for 20~40 s (upper panel). A microsuction-induced nystagmus, if present, was directed towards the contralateral ear (middle panel). In this position, the endolymph in the lateral semicircular canal flows in an ampullofugal direction due to convection (lower panel). (**B**) Upon bowing (upper panel), the nystagmus was directed towards the ipsilateral ear (middle panel). In this position, the endolymph in the lateral semicircular canal flows in an ampullopetal direction due to convection (lower panel). (**C**) Upon leaning (upper panel), the nystagmus was directed towards the contralateral ear (middle panel). In this position, the endolymph in the lateral semicircular canal flows in an ampullofugal direction due to convection (lower panel).

Under an operating microscope, an ear speculum was inserted into the external auditory canal (EAC), and microsuction was performed (Supplemental video S1). The procedure lasted between 20 and 40 s, depending on patient tolerance. Nystagmus was observed during and after microsuction in a seated position. Patients then underwent a bow and lean test, where they bent their heads forward to 90° and tilted backward to 60° (Supplemental video S1, Figure 1), as described previously [25,26,27].

Statistical comparison was performed using IBM SPSS Statistics Data Editor (Ver. 29, Armonk, NY, USA). Categorical variables were compared using the χ^2^ test, and a *p* value < 0.05 was considered to indicate statistical significance.

This study was approved by the Institutional Review Board (approval number: 2024-10-022). The requirement for informed consent was waived by the Institutional Review Board.

## 3. Results

### 3.1. Patients and TM Appearance

Between March 2022 and August 2024, a total of 85 patients were included in this study. The patients were classified based on the appearance of the TM (Table 1). Thirty-five patients exhibited a normal eardrum appearance without TM perforation (normal TM, *n* = 35), seven had previously undergone canal wall-up mastoidectomy with the TM appearing near-normal without re-perforation (normal postoperative TM after canal wall-up mastoidectomy, *n* = 7), and eight patients had otitis media with effusion without TM perforation (otitis media with effusion, *n* = 8). There were 25 patients with TM perforation, of which 14 had chronic suppurative otitis media (chronic suppurative otitis media, *n* = 14), five had cholesteatoma with or without labyrinthine fistula (middle ear cholesteatoma with/without labyrinthine fistula, *n* = 5), and six had a ventilation tube inserted (ventilation tube in situ, *n* = 6). Additionally, six patients had no TM perforation but had a history of open cavity mastoidectomy (TM without perforation after open cavity mastoidectomy, *n* = 6), and four had adhesive otitis media (adhesive otitis media, *n* = 4) (Table 1).

### 3.2. Microsuction-Induced Nystagmus and Vertigo

We investigated the proportion of patients who exhibited nystagmus or complained of vertigo during aural microsuction based on TM appearance. Nystagmus was observed during and after aural microsuction while the patients were seated, and 95% (81 of 85) exhibited microsuction-induced nystagmus (Table 1). The nystagmus was directed toward the contralateral ear (Figure 1A; Supplemental video S1). To determine if the mechanism behind microsuction-induced nystagmus was related to the convection of cooled inner ear fluid, a bow and lean test was subsequently performed. In the bowing position, the direction of nystagmus changed toward the ipsilateral ear (Figure 1B; Supplemental video S1). In the leaning position, the direction of nystagmus reversed and was directed toward the contralateral ear (Figure 1C; Supplemental video S1). Despite the observation that 95% (81 of 85) of patients undergoing microsuction exhibited nystagmus, only 36% (31 of 85) reported experiencing rotatory vertigo (Table 1). Among those who did not report vertigo, none complained of nonspecific dizziness.

Next, we explored whether the proportion of patients who exhibited microsuction-induced nystagmus or complained of vertigo differed according to TM appearance. The results are summarized in Table 1. TM appearance was classified into four groups: (1) No TM perforation group (*n* = 50), including normal TM (*n* = 35), normal postoperative TM after canal wall-up mastoidectomy (*n* = 7), and otitis media with effusion (*n* = 8); (2) TM perforation group (*n* = 25), including chronic suppurative otitis media (*n* = 14), middle ear cholesteatoma with/without labyrinthine fistula (*n* = 5), and ventilation tube in situ (*n* = 6); (3) TM without perforation after open cavity mastoidectomy (*n* = 6); and (4) adhesive otitis media (*n* = 4). The proportion of patients showing nystagmus and reporting rotatory vertigo was 97% (34 of 35) and 9% (3 out of 35), respectively, in patients with normal TM; 100% (seven out of seven) and 29% (two out of seven), respectively, in patients with normal postoperative TM after canal wall-up mastoidectomy; and 63% (five out of eight) and 0%, respectively, in patients with otitis media with effusion. Among patients with chronic suppurative otitis media, 100% (14 out of 14) showed nystagmus and 64% (9 out of 14) reported vertigo. Similarly, all patients with middle ear cholesteatoma with/without labyrinthine fistula (100%, five out of five) showed both nystagmus and vertigo. For those with a ventilation tube in situ, 100% (six out of six) exhibited nystagmus, and 50% (three out of six) experienced vertigo.

**Figure 2:**
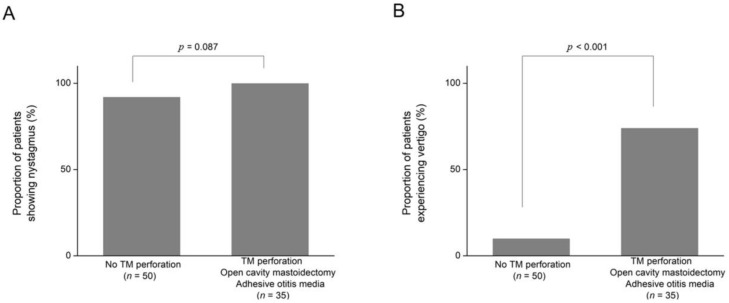
The proportion of patients showing microsuction-induced nystagmus or vertigo according to the appearance of the tympanic membrane (TM). (**A**) The proportion of patients with microsuction-induced nystagmus was higher in patients with normal TM, normal postoperative TM after canal wall-up mastoidectomy, and otitis media with effusion than those with TM perforation, open cavity mastoidectomy, and adhesive otitis media, which was not statistically significant (*p* = 0.087). (**B**) The proportion of patients with microsuction-induced nystagmus was higher in patients with normal TM, normal postoperative TM after canal wall-up mastoidectomy, and otitis media with effusion than those with TM perforation, open cavity mastoidectomy, and adhesive otitis media, which was not statistically significant (*p* = 0.087).

For patients who had undergone open cavity mastoidectomy, 100% (six out of six) showed nystagmus, and 83% (five out of six) reported vertigo. All patients with adhesive otitis media (100%, four out of four) experienced both nystagmus and vertigo (Table 1). The proportion of patients with microsuction-induced nystagmus was not significantly different between the no TM perforation group (92%, 46 out of 50) and the groups with TM perforation, open cavity mastoidectomy, or adhesive otitis media (100%, 35 of 35) (*p* = 0.087, X^2^ test; Figure 2A). However, the proportion of patients reporting rotatory vertigo was significantly higher in the groups with TM perforation, open cavity mastoidectomy, or adhesive otitis media (74%, 26 of 35) compared to the no TM perforation group (10%, 5 of 50) (*p* < 0.001, X^2^ test; Figure 2B).

## 4. Discussion

The present study described the characteristics of nystagmus and the incidence of vertigo in 85 patients who underwent aural microsuction. While microsuction-induced nystagmus was observed in most patients regardless of TM appearance, the proportion of patients reporting vertigo varied depending on the condition of the TM.

It has long been established that a change in temperature in the EAC can cause vertigo and nystagmus [28]. The mechanism of this caloric nystagmus was relatively well established in the original study [28]. The caloric response refers to a physiological reaction of the vestibular system triggered by temperature changes in the EAC. The mechanism of the caloric response involves several steps as follows: (1) Temperature-Induced Changes in Endolymph Dynamics. When warm or cold water/air is introduced into the external auditory canal, it alters the temperature of the nearby horizontal semicircular canal, affecting the movement of endolymph. This temperature shift modifies the density of the endolymph, generating convective currents that simulate head movement. (2) Endolymph Flow and Vestibular Nerve Stimulation. Warm irrigation causes the endolymph to expand and rise due to reduced density. This mimics ampullopetal flow (movement toward the utricle), leading to excitation of the vestibular nerve. Cold irrigation makes the endolymph denser and sink due to thermal contraction. This mimics ampullofugal flow (movement away from the utricle), resulting in the inhibition of the vestibular nerve. These changes in vestibular nerve activity create a neural response that the brain interprets as head rotation, triggering the vestibulo-ocular reflex (VOR) and leading to nystagmus [28,29,30,31,32,33,34,35].

Microsuction during aural toilet lowers the temperature of the EAC and middle ear cavity, which increases the density of the endolymph in the lateral semicircular canal. This change in endolymph density causes convection, leading to excitation or inhibition of the lateral semicircular canal. In 1928, Dundas-Grant reported that suction using Siegel’s speculum produced giddiness accompanied by nystagmus, though it was not always clear [6]. Gray and Nicolaides demonstrated that temperature drops during microsuction, measured using a thermocouple wire, were pronounced in patients experiencing vertigo, especially when suction was applied to ‘wet’ cavities. They suggested that evaporative cooling was the cause. They studied microsuction-induced vertigo in patients with mastoid cavities communicating with the EAC, finding that 80% (16 out of 20) complained of rotatory vertigo with nystagmus, with the fast phase directed toward the opposite ear. Two patients (10%) reported nonspecific dizziness without nystagmus, and two others (10%) reported no dizziness at all [7]. In addition to the evaporative cooling effect as one of main causes of microsuction-induced vertigo, alternative mechanisms including direct mechanical stimulation of the round window, middle ear pressure fluctuations affecting the vestibular system, and psychological factors may also contribute to the production of vertigo during the procedure.

In our study, microsuction-induced nystagmus was observed in 95% (81 out of 85) of patients with various TM appearances. When comparing only patients with a history of open cavity mastoidectomy, the proportion of patients with microsuction-induced nystagmus was higher in our study (100%) compared to the previous study (80%) [7]. This difference may be explained by the use of video Frenzel glasses in our study, as opposed to naked-eye observation in the previous study. Nystagmus can be suppressed by visual fixation, which would occur in naked-eye assessments. It is noteworthy that the proportion of patients with microsuction-induced nystagmus was not significantly different between the no TM perforation group (92%, 46 out of 50) and the groups including TM perforation, open cavity mastoidectomy, and adhesive otitis media (100%, 35 out of 35) in our study. Another interesting finding was that the direction of microsuction-induced nystagmus reversed between the bowing and leaning positions. Considering the anatomy of the lateral semicircular canal in these positions (Figure 1B,C), this supports the hypothesis that microsuction-induced nystagmus is caused by convection of the lateral semicircular canal endolymph due to the cooling effect. We believe that the cooling effect might not be strong enough to induce nystagmus in some patients with otitis media with effusion (Table 1).

While microsuction elicited nystagmus in most patients, only some reported vertigo. A previous study reported that vertigo was experienced by 80% of patients with prior open cavity mastoidectomy during microsuction [10]. In our study, 83% (five out of six) of such patients reported vertigo, which is comparable to previous findings [7]. Notably, vertigo occurred in all patients with adhesive otitis media (four out of four) and middle ear cholesteatoma with/without labyrinthine fistula (five out of five), likely due to more effective transmission of the cooling effect to the lateral semicircular canal. In contrast, vertigo was reported by only 9% of the patients with normal TM, 29% of those with normal postoperative TM after canal wall-up mastoidectomy, and none of the patients with otitis media with effusion. We believe that the cooling effect was less effectively transmitted in patients with adhesive otitis media or previous open cavity mastoidectomy. Additionally, since vertigo is a subjective symptom, the same vestibular stimulus may or may not cause a patient to report vertigo, depending on their sensitivity to symptoms or individual disposition. However, because we did not assess nystagmus intensity in this study, we cannot rule out the possibility that stronger nystagmus could have caused vertigo in some patients, although there is no evidence that the intensity of nystagmus is correlated with the severity of vertigo.

Since the temperature drop during microsuction may cause devastating vertigo in some patients, preventive recommendations to mitigate vertigo during the procedure are to be considered. Although it is difficult to completely eliminate microsuction-induced nystagmus and/or vertigo, the following methods can be considered to alleviate vertigo: (1) The patient’s head is bent forward by approximately 30° in the seated position during the procedure. Considering that the lateral semicircular canal is uptilted anteriorly by approximately 30° in the upright seated position, the horizontal nystagmus, due to the cooling effect during microsuction, would be weakened, resulting in the mitigation of vertigo if the patient bends the head forward by approximately 30°. (2) As suggested by Nicolaides and Gray [9], the warm humified air can be perused during microsuction to minimize the temperature change in the mastoid cavity.

The limitations of this study include the following: (1) It is likely that a consistent vestibular stimulus was not applied to each patient, as the position of the microsuction tip within the EAC and the duration of the procedure may have varied between patients. (2) We investigated the presence of nystagmus but not its intensity, so it remains unclear how the intensity of nystagmus is influenced by TM appearance and its correlation with vertigo symptoms.

## 5. Conclusions

Aural toilet using microsuction commonly induces nystagmus, caused by convection of the lateral semicircular canal endolymph due to the cooling effect. Although microsuction-induced nystagmus was observed in most patients regardless of TM appearance, the percentage of patients reporting vertigo varied according to the condition of the TM. Since some patients may experience severe vertigo during microsuction, which could result in premature termination of the procedure, it is recommended that clinicians closely monitor patients for vertigo during the procedure. Further research is needed to explore methods for preventing microsuction-induced vertigo.

## Figures and Tables

**Table 1:** Proportion of patients with vertigo or nystagmus by aural microsuction according to the appearance of the tympanic membrane (TM).

Appearance of TM (*n* = 85)	Patients Showing Nystagmus (Number of Patients, %)	Patients Experiencing Vertigo (Number of Patients, %)
No TM perforation		
Normal TM (*n* = 35)	34 (97%)	3 (9%)
Normal postoperative TM after canal wall-up mastoidectomy (*n* = 7)	7 (100%)	2 (29%)
Otitis media with effusion (*n* = 8)	5 (63%)	0 (0%)
TM perforation		
Chronic suppurative otitis media (*n* = 14)	14 (100%)	9 (64%)
Middle ear cholesteatoma with/without labyrinthine fistula (*n* = 5)	5 (100%)	5 (100%)
Ventilation tube in situ (*n* = 6)	6 (100%)	3 (50%)
TM without perforation after open cavity mastoidectomy (*n* = 6)	6 (100%)	5 (83%)
Adhesive otitis media (*n* = 4)	4 (100%)	4 (100%)

## Data Availability

Data in this study are available upon request to the corresponding author.

## Supplementary Materials

The following supporting information can be downloaded at https://www.mdpi.com/article/10.3390/audiolres15020033/s1, Supplemental video S1. Representative video of Frenzel glasses examination. Microsuction was applied to the right ear, and left-beating nystagmus was observed. Right-beating nystagmus was observed in a bowing position, and left-beating nystagmus was observed in a leaning position.

## Abbreviations

The following abbreviations are used in this manuscript:

TM	Tympanic membrane
EAC	External auditory canal
